# Metachronous Osseous Metastases From Gliobliostoma Mutiforme: An Unusual Presentation

**DOI:** 10.7759/cureus.22587

**Published:** 2022-02-25

**Authors:** Ajay S Krishnan, Sweety Gupta, Shreyosi Mandal, Ravi H Phulware, Manoj Gupta

**Affiliations:** 1 Department of Radiation Oncology, All India Institute of Medical Sciences, Rishikesh, Rishikesh, IND; 2 Department of Pathology, All India Institute of Medical Sciences, Rishikesh, Rishikesh, IND

**Keywords:** metastatic gbm management, weiss criteria, metachronous metastatic gbm, extracranial metastasic gbm, metastatic gbm

## Abstract

Metastasis occurs very rarely in glioblastoma cases. Diagnosing metastatic glioblastoma has to follow a strict protocol to avoid misdiagnosis. Weiss’s 1955 criteria, which is in prevalence, needs to be modified to include current standards of investigation. We report an interesting case of metachronous osseous metastasis from a primary glioblastoma with a complete response at a local site. We also suggest modifications to Weiss’ criteria, which may improve its utility in establishing the diagnosis of metastatic glioblastoma.

## Introduction

Since glioblastoma multiforme (GBM) was first described in 1864 by Virchow [1], this group of tumours have remained the most common primary malignant tumour of the central nervous system in adults. It constitutes more than one-third of all primary brain tumours [2]. Gliomas rarely metastasize, although they are locally aggressive tumours. Extracranial metastasis is much rarer, accounting for only around 0.2% - 0.3% of cases [1]. Even after the first case of metastatic GBM was reported as early as 1928 by Davis [1], only around 200 patients have been reported worldwide [3], as of 2021. Bone (24%) is the most common site of extracranial metastasis from GBM, followed by lung (22%), lymph nodes (12%), neck (9%), liver (9%) and other systemic sites (24%) [1].

We report a case of osseous metastasis from primary GBM in the absence of local relapse, considering its rarity, and the need for more cases to get an insight into the tumour biology of metastatic GBM.

## Case presentation

**Figure 1:**
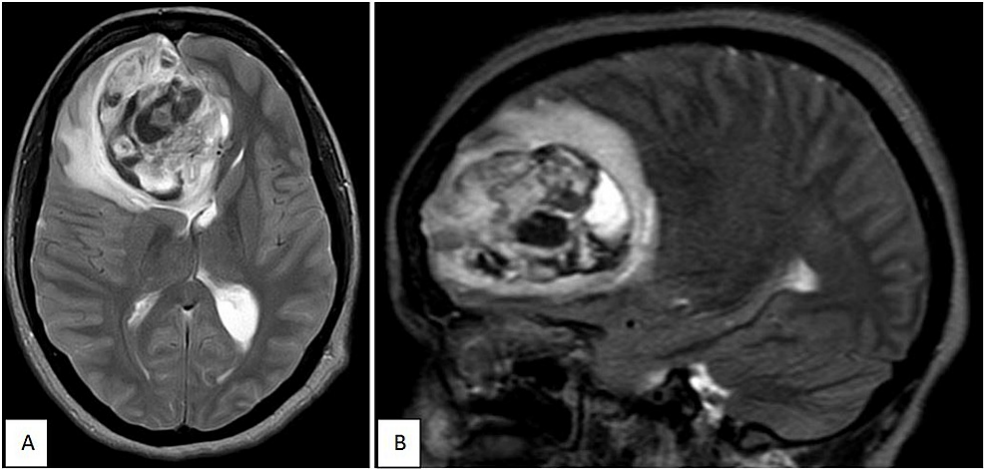
MRI Brain (T2W) - 6.5 x 5.0 cm heterogeneous, T2 hyperintense lesion in the right frontal lobe, involving and effacing surrounding parenchyma, corpus callosum with mild perilesional oedema. There was a midline shift of 10 mm to the left.

A 36-year-old female presented with a single episode of loss of consciousness one week before presentation, along with intermittent, generalised headache and a few episodes of vomiting over one week. She had a positive family history on her maternal side with one first-degree relative, three second-degree relatives and two third-degree relatives having abdominal malignancy. MRI Brain revealed a 6.5 x 5.0 cm T1 isointense and T2 hyperintense lesion in the right frontal lobe, involving and effacing surrounding parenchyma, corpus callosum with mild perilesional oedema. There was a midline shift of 10 mm to the left. The lesion was provisionally diagnosed as a subacute haemorrhage with mass effect (Fig. 1).

The patient underwent a right frontal craniotomy and a near-total excision of the lesion was performed. An ill-defined, highly vascular, greyish, suckable growth was seen in the right frontal lobe involving the corpus callosum and extending to the left side. A histopathological examination of the lesion showed polymorphous tumour cells, arranged in sheets, with round nuclei having coarse chromatin and clear cytoplasm. Brisk mitotic activity was observed. Gemistocytic cells and spindle cells were also seen. Microvascular proliferation and necrosis were seen but no calcifications were observed, consistent with glioblastoma multiforme. In molecular analysis, O6-methylguanine-methyltransferase (MGMT) promoter methylation was not detected; IDH1 and IDH2 (isocitrate dehydrogenase) were not mutated. Also, it was negative for 1p-19q codeletion.

She was started on adjuvant chemoradiotherapy (CCRT). Contouring was done following European Organization for Research and Treatment of Cancer (EORTC) guidelines [4]. She received external beam radiotherapy by Intensity-modulated radiation therapy (IMRT) and concurrent temozolomide. She further received six cycles of adjuvant temozolomide. Because of minimal toxicity, the patient was continued on temozolomide for six more cycles. Follow-up MRI showed no intracranial residual lesion. After the completion of adjuvant chemotherapy, she complained of right-sided hip pain. A bone scan showed multiple osteolytic lesions involving multiple cervical, thoracic, lumbosacral vertebrae; left clavicle, left seventh rib, bilateral scapula, pelvic bones and proximal femora. CT thorax and abdomen showed multiple skeletal metastases and a diffuse enlargement of the thyroid gland.

**Figure 2:**
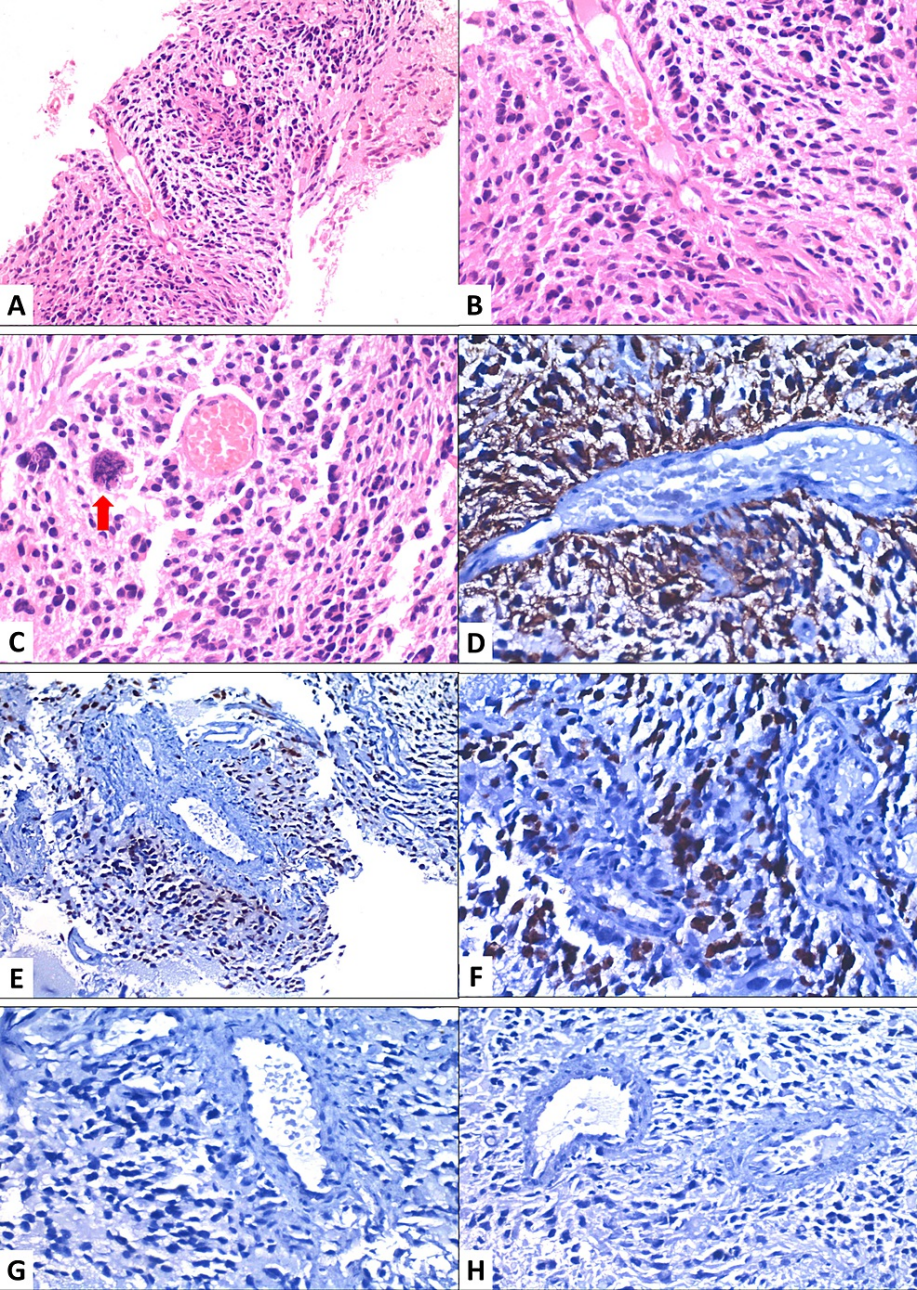
Biopsy from the metastatic lesion in the iliac blade A&B- Biopsy shows viable tumor cells arranged around the blood vessels along with scant tumor necrosis at the periphery (40X & 100X); C- Higher magnification shows nuclear pleomorphism with atypical mitosis (red arrow) (400X); D- Tumor cells expressing GFAP (glial fibrillary acidic protein) immunohistochemistry (400X); E- Tumor cells expressing strong P53 overexpression (200X) F- Ki67 showing increased proliferative activity (400X); G&H- Tumor cells immunonegative for pan cytokeratin and TTF-1 (400X)

**Figure 3:**
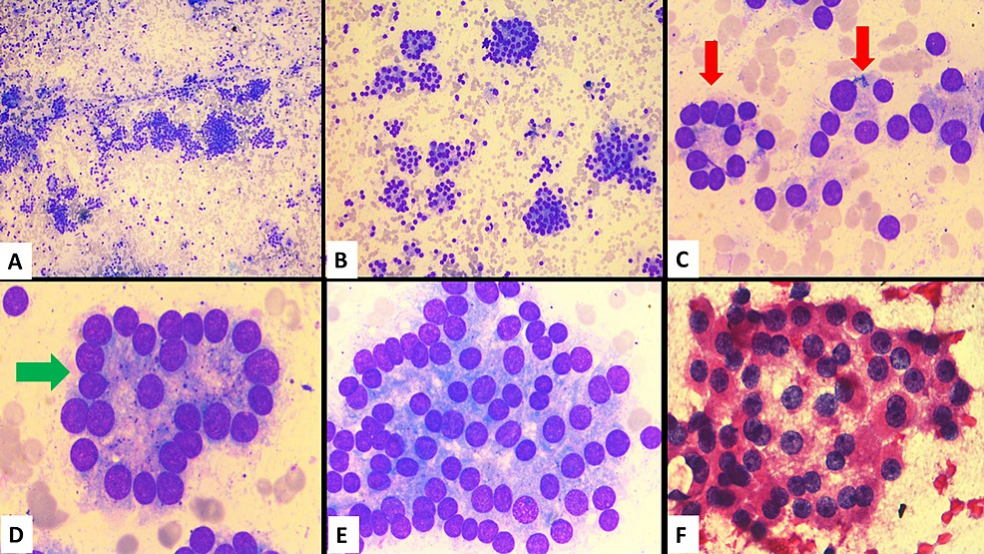
Fine needle aspiration cytology (FNAC) from thyroid A- Low magnification shows cellular smears (40X); B- Higher magnification shows follicular epithelial cells arranged back to back in sheets, micro- and macrofollicles (100X); C- Shows thyroid microfollicles (6-12 nuclei) (red arrow); (400X) D- Shows thyroid macrofollicles (green arrow) (1000X); E and F- Shows sheets of follicular epithelial cells (1000X)

Biopsy from the right iliac blade lesion reported metastatic glioblastoma multiforme. Immunohistochemistry (IHC) showed diffuse positivity of GFAP (glial fibrillary acidic protein), p53 positivity and a Ki67 labelling index of 15% - 20%. In addition, there was a loss of expression of ATRX, and IHC analysis was negative for PanCK, CK-7, CDX-2 (Fig. 2). Fine needle aspiration cytology (FNAC) was done from the thyroid lesion which showed follicular neoplasm (Fig. 3). Suspecting metastasis from the thyroid, IHC analysis for TTF-1 was also done on the specimen from the right iliac blade, which showed a negative result (Fig. 2).

The patient was planned for palliative radiation to the symptomatic bone metastases followed by bisphosphonates and combination chemotherapy with bevacizumab and irinotecan.

## Discussion

The rarity of extracranial metastases in GBM (0.4% - 0.5% of all GBM cases) is hypothesised to be due to two major reasons. First, the survival of GBM was low giving a little window for any metastatic disease to present [3,5]. The median survival as noted by the initial RPA (recursive partitioning analysis) in 1993 was as low as 4.6 - 17.9 months (depending on the RPA class). This hypothesis is supported by the fact that the number of reported cases per decade has been increasing over time [6] due to the improvement in the survival of patients, secondary to the development of better surgical, radiation techniques and the use of chemotherapy. Second, the physical barriers restrict any communication between the intracranial and the extracranial compartment - including the dura mater, the thick basement membrane, and the blood-brain barrier. Also, there is no direct communication between the intracranial (glymphatic system) and extracranial lymphovascular spaces [3]. Finding offsetting this hypothesis include the presence of circulating tumour cells (CTCs), which are found in 20% - 40% of all GBM patients (depending on the method used to identify them) [7,8]. Other hypothesised reasons for the rarity of extracranial metastases in GBM allude to the intrinsic property of the glial filaments, lack of specific extracellular matrix proteins in the extracranial mesenchymal tissue, and the role of the immune system in eliminating the extracranial tumour cells [3].

Even with these temporal, physical and molecular hurdles, there are a few cases that develop metastases outside the CNS. They are believed to occur by haematogenous spread (neovascularisation caused by the growth of the tumour mediated by vascular growth factors; potential accentuation by radiation by increasing vascular permeability, promoting neovascularisation [3,9]); lymphatic spread (invasion of intradural lymphatics); local invasion of disease to extend beyond the physical barriers; invading areas with good lymphatic drainage; sarcomatous metaplasia of the GBM tumour cells [10]; spontaneously acquired mutations, which may be radiation induced.

Establishing the diagnosis as metastatic GBM has to be done carefully since GBM is observed to have low rates of metastasis. Weiss, as early as 1955 [11], had laid out criteria to reasonably ascertain this. This includes four prerequisites: i) Histologically characteristic single tumour in the CNS; ii) Clinical history corroborative of this as the primary tumour; iii) Complete autopsy to be done to rule out any other potential primary lesion; and iv) Morphology of the primary CNS lesion and the metastatic lesion to be identical with due allowance for variation in the degree of anaplasia.

We find that our case fits Weiss's criteria to be labelled as metastatic GBM except for the autopsy criteria. However, it is suitable to note that science has progressed so much that we have other methods to satisfactorily rule out the presence of other primaries. Reviewing the Weiss criteria in the current timeframe, it seems prudent to modify the criteria keeping the essence intact. We suggest the following modifications to the Weiss criteria:

i) To remain as such - Surgery still remains the primary management of GBM. A histological diagnosis remains mandatory for clarity of diagnosis.

ii) To remain as such - Clinical history should be able to provide supportive evidence for the intracranial lesion to temporally precede the metastatic disease.

iii) A whole-body imaging, preferably contrast-enhanced computed tomography (CECT) scan, PET-CT or PET-MRI to rule out any other associated primary disease.

iv) Histological features of the primary and the metastatic disease to be similar and immunohistochemical analysis of the metastatic disease to be suggestive of glial lineage.

Our patient was in her late thirtieswith significant family history. She falls into the Radiation Therapy Oncology Group (RTOG) RPA (Recursive Partitioning Analysis) Class III, putting her expected survival at around 17.1 months (which she has outlived) [12]. The IHC finding from the metastatic site showed p53 mutation in tumour cells. p53 mutations are usually associated with low-grade gliomas and form a part of the spectrum of mutation acquired during their transformation into higher grade gliomas (secondary GBM). However, she does not have a history of a low-grade glioma (grade IV features seen at primary presentation), thus making this a primary GBM. But, speculations persist as to whether any particular mutation or a profile of mutation can predict the development of metastasis in GBM. Routine molecular analysis of the primary tumour specimen needs to be done for any correlation studies to be done in this respect. Currently, no such correlation is documented in the literature.

Another point of interest in the present case was that there was no local relapse even 20 months post-surgery. In conjunction with the fact that there is no gross disease elsewhere apart from the metastatic lesion. This means that the disease had already metastasized when she had received treatment for the local disease. Neither did she have any complaints at the metastatic sites initially nor did she undergo any imaging of the rest of the body to help us temporally pinpoint these metastases. Considering that the metastasis is likely to have been present initially, it is remarkable that she has survived 20 months.

Reports of a better prognosis of GBM with extracranial metastases compared to GBM with intracranial metastases are present in literature [6]. Also, the median age of GBM with extracranial metastases falls around 38 - 42 years [6,13], as against the median age of around 56 years overall in GBM patients (with and without metastases). These form strong proponents for considering aggressive treatment options over best supportive care in such patients whenever clinically feasible. It has also been observed by Lun et al. that an aggressive approach involving surgery, radiotherapy, chemotherapy and cerebrospinal fluid (CSF) shunting gives the best survival outcomes beyond the diagnosis of metastasis (6.1 months) when compared with surgery alone (1.1 months), radiation alone or surgery with radiation (3 months), or surgery with radiotherapy and chemotherapy (4.2 months) [13].

Treatment options

At primary presentation, irrespective of whether the patient presents with metastatic disease or not, surgery followed by adjuvant chemoradiotherapy (which is the standard of care) to the intracranial lesion (CSF shunting to be done when required) and a combination of these modalities at the metastatic site as deemed fit, may be offered if the functional status of the patient is good. Hypofractionated radiotherapy or temozolomide alone may be considered for older patients (70 years or more) or those with poor general condition (Karnofsky performance status < 60 or Eastern Cooperative Oncology Group > 3). When patients present with metastasis (at primary presentation or during follow-up), treatment should be offered with palliative intent. However, more aggressive therapy may be offered as against metronomic therapy, which has been the norm [13].

Temozolomide may be attempted in the recurrent setting and continued up to 12 months or until progression, monitoring for side effects. It may be continued in the conventional dosing schedule (four-weekly schedule with 150 - 200 mg/m2 given D1-5). Other dosing schedules may also be attempted.

Bevacizumab had been approved for use in recurrent GBM based on the BRAIN trial (bevacizumab alone and in combination with irinotecan in recurrent glioblastoma) [14,15]. Use of bevacizumab as monotherapy or in addition to other chemotherapy in the recurrent setting increases the 6-month progression-free survival (PFS) from 9% - 15% (without bevacizumab) to 40% - 50% (with bevacizumab) [15]. Irinotecan with bevacizumab has been attempted in two-weekly as well as six-weekly regimens in recurrent lioblastoma [14,16]. In our study, we used this combination because it gives a six-month PFS advantage of around 8% and a six-month ORR (objective response rate) advantage of around 10% over bevacizumab alone [14]. This is, however, phase II data and did not show a significant overall survival difference. Lomustine alone or in combination with bevacizumab is a reasonable option with phase II studies showing the combination regime have a nine-month overall survival of 43% with lomustine alone, 38% with bevacizumab alone and 59% with a combination of both [17].

Procarbazine, lomustine and vincristine (PCV) chemotherapy has also been tried in the recurrent setting (post-progression on temozolomide) [18]. Regorafenib is another drug studied for its utility in recurrent GBM. According to phase II studies, both have higher toxicities; PCV has lower survival rates, regorafenib may have a benefit over lomustine [19,20].

All treatment options described here are studied in recurrent localised GBM and not in a metastatic setting. But due to the rarity of metastasis in GBM and the unavailability of established guidelines in the metastatic setting, this management may be extrapolated to a metastatic setting.

## Conclusions

Better treatment techniques and options have afforded improved survival for GBM patients. This has clinically translated to a higher proportion of them presenting with metastases. Any complaints of GBM patients are to be evaluated with the possibility of metastasis in mind.

Consider aggressive treatment of GBM with metastasis taking into account the age, the functional status of the patient, since aggressive management may have a survival advantage. Bevacizumab is the common factor among regimens in the recurrent setting which can give maximum benefit. Bevacizumab based chemotherapy is to be considered in the recurrent setting. To put things in perspective, the best available care after recurrence may provide an overall survival that is comparable to an RPA Class V GBM. In the absence of any recommendations, metastatic GBM may be managed following the principles of recurrent gliomas as is described in this report and review.
